# A global bionomic database for the dominant vectors of human malaria

**DOI:** 10.1038/sdata.2016.14

**Published:** 2016-03-01

**Authors:** N. Claire Massey, Gala Garrod, Antoinette Wiebe, Andrew J. Henry, Zhi Huang, Catherine L. Moyes, Marianne E. Sinka

**Affiliations:** 1 Spatial Ecology & Epidemiology Group, Wellcome Trust Centre for Human Genetics, University of Oxford, Oxford, OX3 7BN, UK; 2 Spatial Ecology & Epidemiology Group, Department of Zoology, University of Oxford, Oxford, OX1 3PS, UK

**Keywords:** Behavioural ecology, Ecological epidemiology, Malaria, Entomology

## Abstract

*Anopheles* mosquitoes were first recognised as the transmitters of human malaria in the late 19th Century and have been subject to a huge amount of research ever since. Yet there is still much that is unknown regarding the ecology, behaviour (collectively ‘bionomics’) and sometimes even the identity of many of the world’s most prominent disease vectors, much less the within-species variation in their bionomics. Whilst malaria elimination remains an ambitious goal, it is becoming increasingly clear that knowledge of vector behaviour is needed to effectively target control measures. A database of bionomics data for the dominant vector species of malaria worldwide has been compiled from published peer-reviewed literature. The data identification and collation processes are described, together with the geo-positioning and quality control methods. This is the only such dataset in existence and provides a valuable resource to researchers and policy makers in this field.

## Background and Summary

The behaviour and life history characteristics of a mosquito vector contribute to the relative importance of the species in terms of human malaria transmission^1^. Biting location, biting time and host preference will influence how effectively a mosquito can transmit malaria. In addition, understanding the behaviour of the vector guides how best it can be controlled and the likelihood that a particular intervention measure will be successful^2^. For example, a night feeding, anthropophilic, endophagic and endophilic mosquito (i.e., a vector that preferentially bites humans indoors during the night when people are asleep and vulnerable and then rests indoors) is likely to be a highly effective transmitter of malaria (e.g., *An. funestus*^3^). These same characteristics make this vector an ideal candidate for indoor insecticide-based control such as indoor residual spraying (IRS), which targets mosquitoes that preferentially rest indoors, or long-lasting insecticide-treated nets (LLINs), which target those species attracted to humans indoors at night. On the other hand, a species that is zoophilic, exophagic and exophilic (i.e., a vector that prefers to bite animals and that spends its adult life outdoors) would not be impacted by these control methods (e.g., *An. arabiensis*^3^), but may be vulnerable to outdoor space spraying or insecticidal zooprophylaxis.

Increasingly malaria researchers are turning to transmission models to predict the impact of control measures on malaria transmission, to focus limited resources toward the most efficient measures of control and to address residual transmission^4^. It is becoming more widely accepted that simply scaling up existing insecticide based intervention methods is insufficient to tackle increasingly resistant vector populations or to impact existing, control avoiding species^5–7^. Spatially explicit, species-specific behavioural data are needed to populate the emerging transmission models that aim to identify the pathways to achieve elimination^4^.

**Figure 1:**
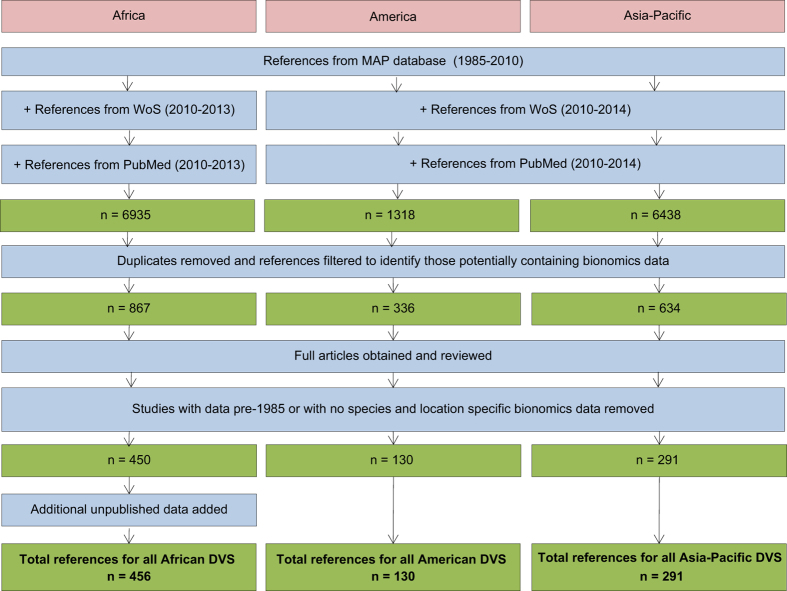
Schematic overview of the data collation and extraction procedure for each region.

The dominant vector species (DVS) of Africa, the Americas and the Asia-Pacific region have previously been identified^1^, and a brief literature survey of vector bionomics was conducted to accompany a series of papers that mapped the ranges of these species^3,8,9^. The survey did not show the proportion of a species showing a particular trait, but instead the proportion of studies reporting the trait for each species. This highlighted two major points. Firstly, a lack of published spatial datasets describing the ecology or behaviour of even the most dominant malaria vectors, and secondly, how much variation in behaviour exists within individual species. A comprehensive search for spatial bionomics data, incorporating behaviour, parasite infection and transmission potential plus other pertinent parameters was therefore conducted (Figure 1).

The focus of this publication is to present the results of this work; a global, species-specific, temporally and spatially categorised database of the bionomics of the DVS of human malaria.

## Methods

Bionomics data were abstracted from the published literature detailing research studies that included data on:

- Vector biology; for example parity and longevity;
- Vector infection and transmission; for example sporozoite rate and entomological inoculation rate;
- Human biting rate;
- Vector host preference (quantifiable measures of anthropo- and zoophily);
- Human biting preference (quantifiable measures of endo- or exophagy);
- Human biting activity (preferred time of biting);
- Resting preference (quantifiable measures of endo- or exophily).

Regional datasets were created for Africa, the Americas and the Asia-Pacific region within which all data were attributed to species and location. There is no single standard method for measuring each of the above parameters so full details including mosquito collection date, season and sampling method were recorded, where given.

### Published data searches

Publications detailing occurrence data for the DVS were identified from the MAP DVS database^8^ (date range of field data: 1985–2010). To ensure an up-to-date dataset, additional searches using the DVS specific names as search terms were conducted in PubMed^10^ and Web of Science^11^ covering literature published from 2010 to May 2013 for the African DVS and August 2014 for the American and Asia-Pacific DVS. Language restrictions were not placed on these searches. Full text digital copies of all publications were obtained. All articles written in English, French, Portuguese and Spanish were read, and those publications with no useful bionomics data were removed. The decision to only include data collected since 1985 was made to ensure that the dataset reflected the current distribution of the DVS and included specimens identified using more up-to-date identification methods and taxonomy^1^.

### Data extraction

Each article was searched for relevant bionomics data related to both a given location and to one of the vector species in question. Data were extracted as reported in the source document, with no assumptions made, and only tabulated data or values reported in the text were accepted (no attempt was made to read graphically reported data from Figures). When possible, bionomics data for individual sibling species were extracted. However, where there was some ambiguity in the species being reported, they were recorded as the species complex (e.g., authors referring to *An. gambiae* but only relying on morphological identification and with no clear indication whether the specimens were considered *An. gambiae* species or *An. gambiae* complex).

In 2013 the *An. gambiae* species was officially split into two formally named species corresponding to the previous classifications of molecular form^12^. Form M is *An. coluzzii* and Form S is *An. gambiae*. Our study began before this classification was published and much of the data collated and included in this current work refers to *An. gambiae* in its old form (i.e., inclusive of *An. coluzzii* and *An. gambiae*). Where given, we captured full species details, however these use the old molecular form classification. Therefore, consider all mentions of *An. gambiae* to include *An. coluzzii* and *An. gambiae* unless specifically stated otherwise. Our dataset also records the previous classification of chromosomal form, where given. On occasion, despite conducting additional identifications to determine sibling species, authors presented their bionomics data for the species complex, this was also recorded as given.

Where possible all data reported in the source for a specific location, time and species are combined on a single data line. For example, this means there may be information relating to a vector population’s host preference, sporozoite infection rate and peak biting time all combined on a single row. However, as not all bionomics parameters were reported by every study this also means that there are blank cells on each row. Blanks cells always represent ‘no data’.

Where given, season was recorded. Due to the high influence of season on mosquito behaviour and abundance, when it was not provided it was calculated from the dates given, either in the source or by searching for information detailing when the rainy and dry seasons normally occurred in the specific location. When season has been calculated this is recorded in a separate column, so that users of the dataset are aware that this was not included in the original data source.

For the African dataset a search of the accumulated bionomics library was conducted to identify those authors who were most prolific in publishing pertinent bionomics data. These authors were contacted to ask if they had any further, unpublished data they would be able to contribute. Any unpublished data was added into the dataset as above. Authors were also contacted to clarify details that were unclear or to disaggregate data where the source suggested more detail may have been collected in the study, but had not been presented within the published source. Due to time constraints this step was not carried out for the American and Asia-Pacific datasets.

### Site geo-positioning

The majority of sites sampled had previously been geolocated in an earlier study mapping the ranges of the DVS^3,8,9^. All additional sites were georeferenced following the same protocol, fully detailed in Hay *et al*^1^. In brief, site location was determined by searching for the site name in online gazetteers (e.g., Google Earth, Google Maps, GeoNames) or other geolocational resources (e.g., Microsoft Encarta). Site related contextual information provided in the original reference (e.g., ‘10 km from the coast’) was used to confirm that the correct site had been identified. Data locations were attributed to area types, including point locations (within 10 km^2^), wide areas (10–25 km^2^), small polygons (25–100 km^2^) or large polygons (>100 km^2^). Single sampling points were identified as point locations. However, data were often reported for several sampling sites combined. In this case, sampling locations were determined as a wide areas, small polygons or large polygons depending on the extent of the sampling area. A single set of coordinates for the most central sampling site of the study are used to define the location of the sampling area, with the area type used to give an indication of the geographic spread of the sampling locations.

### Bionomics datasets

We define a data record as a data point for a unique site-date-species combination. Table 1 reports the total number of data sources reviewed and the number of data records available for the most often reported bionomic parameters. A total of 1,837 published data sources were reviewed and data abstracted from 871 of these. Additional data were incorporated from 6 unpublished sources as a result of author contacts.

**Figure 2:**
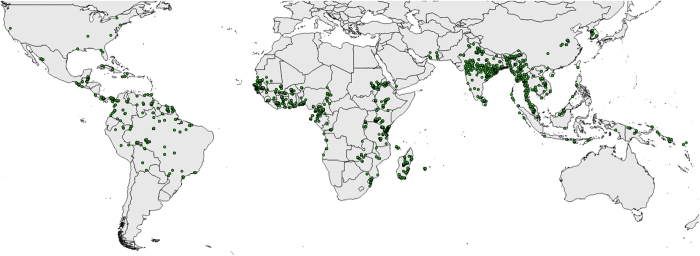
Map of locations with bionomics data. This Figure includes all DVS, regions and bionomic parameters.

Figure 2 shows the locations of the bionomics data study sites and indicates the global spread of the data. A list of countries for which at least one data record is available is shown in Table 2.

**Figure 3:**
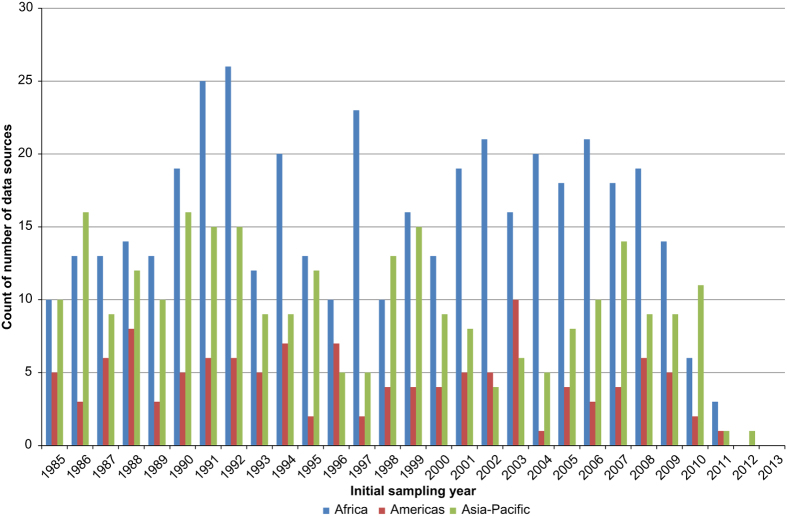
Graph showing the initial sampling year of the bionomic data sources from each region.

Figure 3 displays the temporal spread of the data, by showing the starting year of the sampling period for each data source. There is no clear pattern to the number of published bionomic studies from each year, with a roughly equal spread of studies across time for each region. The lower number of studies for each region from 2010 onwards is possibly due to the lag period between field work and publication of results.

Table 3 presents the number of data sources containing bionomics data for a selection of the most important DVS for malaria transmission, together with the number of individual sampling sites. The number of data records available for these species for a selection of the key parameters is also shown in Table 3. Although the number of data records is summed for members of a species complex in this summary Table, they are recorded separately for each sibling species in the bionomics datasets, if this was reported in the primary data source.

## Data Records

The three regional databases are publicly available online as comma delimited files (Data Citation 1). The data are also available via the Malaria Atlas Project (MAP) website^13^ and the VecNet digital library^14^. Each survey included in the vector bionomics database has been disaggregated to individual sites (geographical points), individual dates (if the same site was sampled repeatedly) and individual *Anopheles* DVS. Values were extracted to a database with the following fields:

### IDENTIFICATION

**Source_ID**. Unique source identifier.

**Country**. Country where the study was conducted.

**Site**. Site name.

**Lat**. Latitude in decimal degrees.

**Long**. Longitude in decimal degrees.

**Area_type**. Point (within 10 km^2^), wide area (10–25 km^2^), small polygon (25–100 km^2^) or large polygon (>100 km^2^).

**Insecticide_control**. Indicates whether insecticide based control methods are in place (previously implemented or implemented as part of the referenced study) at the specified location and time period.

- T: TRUE.
- F: FALSE.
- blank if unknown.

**Control_type**. If ‘TRUE’ above, details the insecticide control method.

- ITN: insecticide treated nets.
- IRS: indoor residual spraying.
- IT curtains: insecticide treated curtains.
- Coil: coil.
- Combination: more than one control method used.
- ?: not stated.

**Month_start**. Survey start month.

**Month_end**. Survey end month.

**Year_start**. Survey start year.

**Year_end**. Survey end year.

**Season_given**. Rainy or dry season at the time of the survey, as indicated in the source.

**Season_calc**. Rainy or dry season at the time of the survey, as derived from information on the general seasonal timings provided from the source or elsewhere.

**Species**. The *Anopheles* species, species complex or subgroup. Also includes molecular form or chromosomal form if reported.

**ASSI**. Additional species-specific information given in the source and provided as a free text field.

**Id_1**. The method used to identify species.

- Chromosome banding: banding patterns on chromosomes.
- Cyto: cytological=cell/chromosomal characteristics.
- DNA: other DNA probing methods without PCR.
- M: morphological.
- Palpal ratio: palpal ratio.
- PCR: Polymerase Chain Reaction amplification techniques.
- Polytene chromosome: banding patterns on polytene chromosomes.
- PCR/DNA: PCR combined with DNA probe.
- Blank: unknown or unreported identification method.

**Id_2**. The second method used to identify species, using same options as above.

### VECTOR BIOLOGY

**Biology_sampling_1**. The sampling methods used to collect the specimens detailed in the VECTOR BIOLOGY section. Three methods can be listed. If more than three methods have been used, this is indicated as ‘t’ in the final column.

- MBI: Human biting indoors
- MBO: Human biting outdoors
- MB: Human biting (location not specified)
- ABI: Animal biting indoors
- ABO: Animal biting outdoors
- AB: Animal biting (location not specified)
- HRI: House resting indoors
- ILT: Indoor light trap
- OLT: Outdoor light trap
- RO: Resting outdoors (location not specified, or locations combined)
- RO (pit): Resting outdoors in pits
- RO (shelter): Resting outdoors in a shelter
- RO (ani-shelter): Resting outdoors in an animal shelter
- WinExit: Window exit traps
- HBN: Human baited net
- ABN: Animal baited net
- Odour-trap: Odour trap
- Tent trap: Tent trap
- Col. Curtains: Colombian curtains
- ?: Sampling method not specified

**Biology_sampling_2.** As ‘Biology_sampling_1’.

**Biology_sampling_3.** As ‘Biology_sampling_1’.

**Biology_sampling_n.** ‘t’ indicates that there are more than three sampling methods.

**Parity_n**. The number of parous females detected from the total number examined.

**Parity_total**. The total number of females examined for parity.

**Parity_percent**. The percentage of parous females in the sample: number of parous females/total number examined*100.

**Daily_survival_rate_percent**. The estimated proportion of female mosquitoes alive on day *d* that are still alive on day *d*+1.

**Fecundity**. The number of eggs laid per batch.

**Gonotrophic_cycle_days**. The number of days for a female mosquito to go through the reproduce-feeding cycle.

### VECTOR INFECTION RATE

**Infection_sampling_1**. The sampling methods used to collect the specimens detailed in the VECTOR INFECTION RATE section. Three methods can be listed. If more than three methods have been used, this is indicated as ‘t’ in the final column. As ‘Biology_sampling_1’.

**Infection_sampling_2.** As ‘Infection_sampling_1’.

**Infection_sampling_3.** As ‘Infection_sampling_1’.

**Infection_sampling_n.** ‘t’ indicates that there are more than three sampling methods.

**SR_dissection_n**. The number of sporozoite infected females detected by dissection from the total number examined.

**SR_dissection _total**. The total number of females dissected for sporozoites.

**SR_dissection_percent**. The percentage of sporozoite infected females detected by dissection in the sample: number of infected females/total number examined*100.

**SR_CSP_n**. The number of sporozoite infected females detected by circumsporozoite protein (CSP) analysis from the total number examined.

**SR_CSP_Pf_n**. The number of *P. falciparum* specific sporozoite infected females detected by CSP analysis from the total number examined. This field is only included for the Americas and the Asia-Pacific region.

**SR_CSP_Pv_n**. The number of *P. vivax* (variant not stated or combined) specific sporozoite infected females detected by CSP analysis from the total number examined. This field is only included for the Americas and the Asia-Pacific region.

**SR_CSP_Pv_210_n**. The number of *P. vivax* variant 210 specific sporozoite infected females detected by CSP analysis from the total number examined. This field is only included for the Americas and the Asia-Pacific region.

**SR_CSP_Pv_247_n**. The number of *P. vivax* variant 247 specific sporozoite infected females detected by CSP analysis from the total number examined. This field is only included for the Americas and the Asia-Pacific region.

**SR_CSP_Pm_n**. The number of *P. malariae* specific sporozoite infected females detected by CSP analysis from the total number examined. This field is only included for the Americas and the Asia-Pacific region.

**SR_CSP_Po_n**. The number of *P. ovale* specific sporozoite infected females detected by CSP analysis from the total number examined. This field is only included for the Americas and the Asia-Pacific region.

**SR_CSP_total**. The total number of females analysed for CSP.

**SR_CSP_percent**. The percentage of sporozoite infected females detected by CSP analysis in the sample: number of infected females/total number analysed*100.

**SR_CSP_Pf_percent**. The percentage of *P. falciparum* specific sporozoite infected females detected by CSP analysis in the sample: number of *P. falciparum* specific infected females/total number analysed*100. This field is only included for the Americas and the Asia-Pacific region.

**SR_CSP_Pv_percent**. The percentage of *P. vivax* (variant not stated or combined) specific sporozoite infected females detected by CSP analysis in the sample: number of *P. vivax* specific infected females/total number analysed*100. This field is only included for the Americas and the Asia-Pacific region.

**SR_CSP_Pv_210_percent**.The percentage of *P. vivax* variant 210 specific sporozoite infected females detected by CSP analysis in the sample: number of *P. vivax* variant 210 specific infected females/total number analysed*100. This field is only included for the Americas and the Asia-Pacific region.

**SR_CSP_Pv_247_percent**.The percentage of *P. vivax* variant 247 specific sporozoite infected females detected by CSP analysis in the sample: number of *P. vivax* variant 247 specific infected females/total number analysed*100. This field is only included for the Americas and the Asia-Pacific region.

**SR_CSP_Pm_percent**. The percentage of *P. malariae* specific sporozoite infected females detected by CSP analysis in the sample: number of *P. malariae* specific infected females/total number analysed*100. This field is only included for the Americas and the Asia-Pacific region.

**SR_CSP_Po_percent**. The percentage of *P. ovale* specific sporozoite infected females detected by CSP analysis in the sample: number of *P. ovale* specific infected females/total number analysed*100. This field is only included for the Americas and the Asia-Pacific region.

**Oocyst_n**. The number of oocyst infected females detected from the total number examined.

**Oocyst_total**. The total number of females examined for oocysts.

**Oocyst_percent**. The percentage of oocyst infected females detected in the sample: number of infected females/total number examined*100.

**EIR.** The entomological inoculation rate. This is the number of infective bites per person per unit time.

**EIR_period**. The unit of time relating to the EIR.

**Ext_incubation_period_days**. The extrinsic incubation period of the malaria parasite in days.

### HUMAN BITING RATE

**Indoor_HBR_sampling**. The sampling method used to collect the mosquitoes from which indoor human biting rate is evaluated. As ‘Biology_sampling_1’.

**Indoor HBR**. The indoor human biting rate; the number of bites per person per unit time.

**Outdoor_HBR_sampling**. The sampling method used to collect the mosquitoes from which outdoor human biting rate is evaluated. As ‘Biology_sampling_1’.

**Outdoor HBR**. The outdoor human biting rate; the number of bites per person per unit time.

**Combined_HBR_sampling_1**. The sampling methods used to collect the mosquitoes from which human biting rate is evaluated where data are amalgamated from more than one method (e.g., where HBRs are given from combined indoor and outdoor sampling methods, or where the method used is unclear). Three methods can be listed. If more than three methods have been used, this is indicated as ‘t’ in the final column. As ‘Biology_sampling_1’.

**Combined_HBR_sampling_2**. As ‘Combined_HBR_sampling_1’.

**Combined_HBR_sampling_3**. As ‘Combined_HBR_sampling_1’.

**Combined_HBR_sampling_n**. ‘t’ indicates that there are more than three sampling methods.

**Combined_HBR**. The human biting rate evaluated from the data from amalgamated sampling methods.

**HBR_unit**. The unit time for the HBR data.

### VECTOR HOST PREFERENCE

**Indoor_host_sampling**. The indoor sampling method used to collect the mosquitoes from which indoor host preference is evaluated. As ‘Biology_sampling_1’.

**Indoor_host_n**. The number of mosquitoes positively indicating a measure of host preference from the total number collected indoors.

**Indoor_host_total**. The total number of mosquitoes sampled indoors examined for measures of host preference.

**Indoor host**. The measure of host preference from indoor sampled mosquitoes.

**Outdoor_host_sampling**. The outdoor sampling method used to collect the mosquitoes from which outdoor host preference is evaluated. As ‘Biology_sampling_1’.

**Outdoor_host_n**. The number of mosquitoes positively indicating a measure of host preference from the total number collected outdoors.

**Outdoor_host_total**. The total number of mosquitoes sampled outdoors examined for measures of host preference.

**Outdoor host**. The measure of host preference from outdoor sampled mosquitoes.

**Combined_host_sampling_1**. The sampling methods used to collect the mosquitoes from which host preference is evaluated where data are amalgamated from more than one method, or where the method used is unclear. Three methods can be listed. If more than three methods have been used, this is indicated as ‘t’ in the final column. As ‘Biology_sampling_1’.

**Combined_host_sampling_2**. As ‘Combined_host_sampling_1’.

**Combined_host_sampling_3**. As ‘Combined_host_sampling_1’.

**Combined_host_sampling_n**. ‘t’ indicates that there are more than three sampling methods.

**Combined_host_n**. The number of mosquitoes positively indicating a measure of host preference collected by a combination of sampling methods.

**Combined_host_total**. The total number of mosquitoes sampled by a combination of sampling methods, examined for measures of host preference.

**Combined_host**. The measure of host preference from mosquitoes sampled by a combination of methods.

**Host_unit**. Indicates the measure used to identify host preference.

- HBI (%): Human Blood Index as a percentage.
- ABI (%): Animal Blood Index as a percentage.
- HBI (%calc): Human Blood Index as a percentage calculated from data given in source.
- ABI (%calc): Animal Blood Index as a percentage calculated from data given in source.
- AI: ‘Anthropophilic Index’, a measure of attraction to humans not included above, for example % individuals attracted to human baited trap over total collected in both human and cattle baited trap, calculated from count data.

NB. the unit ‘HBI (%calc)’ and ‘ABI (%calc)’ is where the source provides the raw data needed to calculated HBI or ABI but does not actually present these data. The unit indicates that the calculation has been done here.

**Other_host_sampling_1**. The sampling methods used to collect the mosquitoes from which host preference is evaluated where additional data are presented examining host preference. Three methods can be listed. If more than three methods have been used, this is indicated as ‘t’ in the final column. As ‘Biology_sampling_1’.

**Other_host_sampling_2**. As ‘Other_host_sampling_1’.

**Other_host_sampling_3**. As ‘Other_host_sampling_1’.

**Other_host_sampling_n**. ‘t’ indicates that there are more than three sampling methods.

**Other_host_n**. The number of mosquitoes positively indicating a measure of host preference.

**Other_host_total**. The total number of mosquitoes examined for measures of host preference.

**Other_host**. The measure of host preference

**Other_host_unit**. As ‘Host_unit’.

### HUMAN BITING LOCATION AND TIME

**Indoor_number_sampling_nights_biting**. The sampling effort, in number of ‘man nights’, to collect the indoor biting data.

**Indoor_biting_sampling**. The sampling method used to collect the indoor mosquitoes from which biting location preference is determined. As ‘Biology_sampling_1’.

**Indoor_biting_n**. The number of mosquitoes found biting indoors.

**Indoor_biting_total**. The total number of indoor and outdoor biting mosquitoes.

**Indoor_biting**. The percentage or ratio of mosquitoes found biting indoors.

**Outdoor_number_sampling_nights_biting.** The sampling effort, in number of ‘man nights’, to collect the outdoor biting data.

**Outdoor_biting_sampling**. The sampling method used to collect the outdoor mosquitoes from which biting location preference is determined. As ‘Biology_sampling_1’.

**Outdoor_biting_n**. The number of mosquitoes found biting outdoors.

**Outdoor_biting_total**. The total number of indoor and outdoor biting mosquitoes.

**Outdoor_biting**. The percentage or ratio of mosquitoes found biting outdoors.

**Indoor_outdoor_biting_units.** Indicates the data unit for the indoor and outdoor biting data.

- I:O: Indoor to outdoor ratio.
- %: % biting indoors (or outdoors) given in source.
- %calc: % biting indoors (or outdoors) calculated from data given in source.
- NB. the unit ‘%calc’ is where the source provides the raw data for indoor and outdoor biting densities but does not calculate the percentage indoors/outdoors. The unit indicates that the calculation has been done here.

**Indoor_number_sampling_nights_biting_activity**. The sampling effort, in number of ‘man nights’, relevant to indoor biting activity data.

**Indoor_1830_2130**. ‘t’ given here if indoor biting activity peaks in the first quarter of the night, includes dusk biting.

**Indoor_2130_0030**. ‘t’ given here if indoor biting activity peaks in the second quarter of the night.

**Indoor_0030_0330**. ‘t’ given here if indoor biting activity peaks in the third quarter of the night.

**Indoor_0330_0630**. ‘t’ given here if indoor biting activity peaks in the fourth quarter of the night, includes dawn biting.

**Outdoor_number_sampling_nights_biting_activity**. The sampling effort, in number of ‘man nights’, relevant to outdoor biting activity data.

**Outdoor_1830_2130**. ‘t’ given here if outdoor biting activity peaks in the first quarter of the night, includes dusk biting.

**Outdoor_2130_0030**. ‘t’ given here if outdoor biting activity peaks in the second quarter of the night.

**Outdoor_0030_0330**. ‘t’ given here if outdoor biting activity peaks in the third quarter of the night.

**Outdoor_0330_0630**. ‘t’ given here if outdoor biting activity peaks in the fourth quarter of the night, includes dawn biting.

**Combined_number_sampling_nights_biting_activity**. The sampling effort, in number of ‘man nights’, relevant to biting activity data where data are presented for both indoor and outdoor biting combined.

**Combined_1830_2130**. ‘t’ given here if combined biting activity peaks in the first quarter of the night, includes dusk biting.

**Combined_2130_0030**. ‘t’ given here if combined biting activity peaks in the second quarter of the night.

**Combined_0030_0330**. ‘t’ given here if combined biting activity peaks in the third quarter of the night.

**Combined_0330_0630**. ‘t’ given here if combined biting activity peaks in the fourth quarter of the night, includes dawn biting.

### VECTOR RESTING LOCATION PREFERENCE

**Indoor_resting_sampling**. Indoor sampling method used to collect the mosquitoes to assess indoor resting behaviour. As ‘Biology_sampling_1’.

**Indoor_unfed**. Total number of unfed mosquitoes in the sample collected indoors.

**Indoor_fed**. Total number of fed mosquitoes in the sample collected indoors.

**Indoor_gravid**. Total number of gravid mosquitoes in the sample collected indoors.

**Indoor_total**. Total number of mosquitoes in the sample collected indoors, including unfed, fed and gravid females.

**Outdoor_resting_sampling**. Outdoor sampling method used to collect the mosquitoes to assess outdoor resting behaviour. As ‘Biology_sampling_1’.

**Outdoor_unfed**. Total number of unfed mosquitoes in the sample collected outdoors.

**Outdoor_fed**. Total number of fed mosquitoes in the sample collected outdoors.

**Outdoor_gravid**. Total number of gravid mosquitoes in the sample collected outdoors.

**Outdoor_total**. Total number of mosquitoes in the sample collected outdoors, including unfed, fed and gravid females.

**Other_resting_sampling**. Sampling methods relevant to ‘other’ data. These columns are used when additional sampling is reported, for example if indoor and outdoor resting mosquitoes are listed in the previous sections, but the source also reports data from a third sampling method such as mosquitoes resting in animal sheds. As ‘Biology_sampling_1’.

**Other_unfed**. Total number of unfed mosquitoes in the sample collected by additional/‘other’ methods.

**Other_fed**. Total number of fed mosquitoes in the sample collected by additional/‘other’ methods.

**Other_gravid**. Total number of gravid mosquitoes in the sample collected by additional/‘other’ methods.

**Other_total**. Total number of mosquitoes in the sample collected by additional/‘other’ methods, including unfed, fed and gravid females.

**Resting_unit**. The unit relating to the indoor, outdoor or other resting data.

- Count: raw count data.
- %: percentage.
- Per man hour: total number collected divided by time spent collecting in hours.
- Fed:gravid: fed to gravid ratio, total number of fed specimens divided by total number of gravid specimens.

### CITATION

**Citation**. The data source.

**PubMed**_**ID**. PubMed ID, when available.

## Technical Validation

Bionomics data have been recorded by a large number of researchers, often using different sampling methods and reporting the data using different metrics. Due to the complicated and non-standard nature of the data, all data were reviewed and checked by a second data abstractor. The data were also checked to ensure that recorded values were within the possible ranges (for example between 0 and 100 for parameters recorded as percentages) and that all values had associated units.

To ensure all locations were accurately geo-located these were again confirmed by a second data abstractor. As many of the data sources identified in this project had previously been included in mapping projects on parasite rate^15,16^ and vector occurrence^3,8,9^ the geolocation coordinates for these sites had already been confirmed. Coordinates were also plotted to ensure that they fall on land and in the correct country.

## Usage Notes

This is the first time that a comprehensive global database has been compiled of published bionomics data for the DVS of human malaria. The dataset described here will be of value to researchers when assessing the likely impact of vector control measures on malaria transmission and to policy makers when deciding how malaria control resources are allocated. Searching the dataset for data related to a specific DVS, geographic location or bionomic parameter will allow the user to quickly identify the available data, and to link this back to the original data source. In addition, this dataset can be used to identify the current knowledge gaps in the behaviour and life history characteristics of the DVS across their geographic ranges.

The published studies did not use consistent units for each of the parameters of interest, and no attempt has been made to standardise the units as part of this work. It is vitally important that the values for each parameter are not treated as single dataset that used a common methodology and unit. Users are strongly advised to examine the sampling methods and units fields provided for each parameter when making use of the data.

We will be using these data to test specific hypotheses relating to the DVS of Africa, including the presence of an east-west behavioural cline; whether insecticide control has caused a continent-wide non species-specific shift to exophagy amongst previously endophagic species; whether insecticide control has caused a continent-wide non species-specific shift in biting times amongst night biting species; and whether the DVS are really behaviourally flexible, or if the observed plasticity actually relates to different sub-species or sibling species within a complex.

## Additional Information

**How to cite this article:** Massey, N.C. *et al.* A global bionomic database for the dominant vectors of human malaria. *Sci. Data* 3:160014 doi: 10.1038/sdata.2016.14 (2016).

## Supplementary Material



## Figures and Tables

**Table 1:** Summary metrics for the datasets and most commonly reported bionomic parameters.

	**Region**		
	**Africa**	**Americas**	**Asia-Pacific**
Number of papers reviewed	867	336	634
Number of papers with relevant data	450	130	291
Number of unpublished data sources	6	—	—
	Number of data records extracted:		
Parity	1,015	251	742
Sporozoite rate	3,254	269	1,104
Entomological inoculation rate	1,648	23	119
Human biting rate	3,107	691	1,773
Anthropo-/Zoophily	1,124	90	494
Endo-/Exophagy	441	233	242
Endo-/Exophily	340	43	660

**Table 2:** A list of countries for which bionomics data are available.

**Africa**	**Americas**	**Asia-Pacific**
Angola	Belize	Bangladesh
Benin	Bolivia	Cambodia
Burkina Faso	Brazil	China
Burundi	Colombia	India
Cameroon	Costa Rica	Indonesia
Chad	Cuba	Iran
Comoros	Dominican Republic	Republic of Korea
Congo	French Guiana	Lao People’s Democratic Republic
Côte d’Ivoire	Guatemala	Malaysia
Democratic Republic of the Congo	Guyana	Myanmar
Equatorial Guinea	Haiti	Nepal
Eritrea	Mexico	Pakistan
Ethiopia	Panama	Papua New Guinea
Gabon	Peru	Philippines
Ghana	Suriname	Singapore
Guinea	Trinidad and Tobago	Solomon Islands
Guinea-Bissau	United States of America	Sri Lanka
Kenya	Venezuela	Taiwan
Madagascar		Thailand
Malawi		Timor-Leste
Mali		Viet Nam
Mauritania		Yemen
Mozambique		
Niger		
Nigeria		
Réunion		
São Tomé and Príncipe		
Senegal		
Sierra Leone		
South Africa		
Sudan		
Tanzania		
The Gambia		
Uganda		
Zambia		
Zimbabwe		

**Table 3:** Number of data sources, individual sites and data records for the most commonly reported bionomic parameters for a selection of the most important DVS in each region.

			**Number of data records**							
	**Number of data sources**	**Number of sites**	**Parity**	**Sporozoite rate**	**Entomological inoculation rate**	**Indoor human biting rate**	**Outdoor human biting rate**	**Anthropo-/Zoophily**	**Endo-/Exophagy**	**Endo-/Exophily**
Africa										
*An. arabiensis*	137	279	164	537	248	197	180	260	70	75
*An. funestus* *	245	495	234	830	508	375	196	337	145	76
*An. gambiae* †	356	871	551	1,604	794	467	182	472	182	189
Americas										
*An. albitarsis* ‡	47	77	67	69	1	0	67	9	24	3
*An. darlingi*	69	149	32	118	19	18	89	5	73	4
*An. pseudopunctipennis* §	20	35	37	11	0	24	23	42	50	17
Asia-Pacific										
*An. culicifacies* ||	104	298	126	260	10	41	19	195	16	308
*An. dirus* ¶	63	117	149	166	58	111	149	18	26	10
*An. farauti* #	23	65	15	29	8	9	17	13	13	7
*An. fluviatilis* **	50	145	87	121	1	58	26	67	2	95
*An. koliensis*	15	36	4	7	0	13	15	15	3	0
*An. minimus* ††	72	138	91	114	19	116	159	21	71	8
*An. punctulatus* ‡‡	20	42	23	13	1	25	31	11	3	1
*An. stephensi*	27	49	6	57	0	0	0	18	1	29

*These values indicate the total number of data records for *An. funestus* complex and *An. funestus*.

^†^ These values indicate the total number of data records for *An. gambiae* complex, *An. gambiae* (formerly Species A), *An. gambiae* (Forest), *An. gambiae* (Bamako), *An. gambiae* (Savanna), *An. gambiae* (Mopti), *An. gambiae* (Bissau), *An. gambiae* (Form M) and *An. gambiae* (Form S).

^‡^ These values indicate the total number of data records for *An. albitarsis* complex, *An. albitarsis* (formerly Species A), *An. albitarsis* (Species B), *An. marajoara* (formerly Species C), *An. albitarsis* (Species D) and *An. albitarsis* (Species E).

^§^ These values indicate the total number of data records for *An. pseudopunctipennis* complex, *An. pseudopunctipennis* (Species A), *An. pseudopunctipennis* (Species B) and *An. pseudopunctipennis* (Species C).

^||^ These values indicate the total number of data records for *An. culicifacies* complex, *An. culicifacies* (Species A), *An. culicifacies* (Species B), *An. culicifacies* (Species C), *An. culicifacies* (Species D) and *An. culicifacies* (Species E).

^¶^ These values indicate the total number of data records for *An. dirus* complex, *An. dirus* (formerly Species A), *An. cracens* (formerly Species B), *An. scanloni* (formerly Species C), *An. baimaii* (formerly Species D), *An. elegans* (formerly Species E) and *An. nemophilous* (formerly Species F).

^#^ These values indicate the total number of data records for *An. farauti* complex, *An. farauti* (formerly No. 1), *An. hinesorum* (formerly No. 2), *An. farauti* (No. 4), *An. farauti* (No. 5), *An. farauti* (No. 6), *An. farauti* (No. 7) and *An. farauti* (No. 8).

**These values indicate the total number of data records for *An. fluviatilis* complex, *An. fluviatilis* (Species S), *An. fluviatilis* (Species T) and *An. fluviatilis* (Species U).

^††^ These values indicate the total number of data records for *An. minimus* complex, *An. minimus* (formerly Species A), *An. harrisoni* (formerly Species C), and *An. yaeyamaensis* (formerly Species E).

^‡‡^ These values indicate the total number of data records for *An. punctulatus* complex and *An. punctulatus*.
